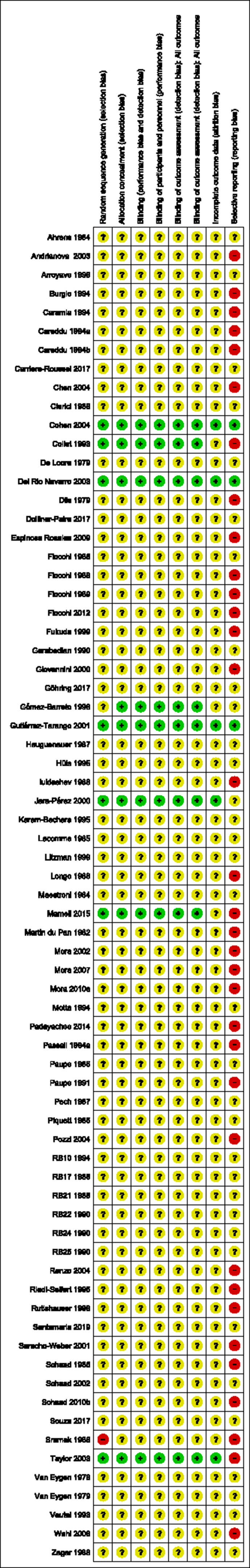# Immunostimulants for preventing respiratory tract infection in children: A systematic review and meta-analysis

**DOI:** 10.1016/j.waojou.2022.100684

**Published:** 2022-09-14

**Authors:** Arturo Berber, Blanca Estela Del-Río-Navarro, Nayely Reyes-Noriega, Juan José Luis Sienra-Monge

**Affiliations:** a External Collaborator of the Hospital Infantil de México Federico Gómez, Mexico; b Allergy and Immunology Department of the Hospital Infantil de México Federico Gómez, Mexico

**Keywords:** Respiratory tract infections, Immunostimulants, Children, Prevention, Safety, Efficacy

## Abstract

Childhood acute respiratory tract infections (ARTIs) are a significant cause of morbidity and mortality, so, immunostimulants have been used as a preventative measure. Despite this, there is no updated evidence regarding the safety and efficacy of immunostimulant drugs for this purpose. This study aimed to determine the effectiveness and safety of immunostimulants in preventing ARTIs in children based on the most recent scientific evidence. Data sources such as PubMed, Cochrane Central Register of Controlled Trials, Embase, Google Scholar, and Scopus were searched from 1965 to 10 January 2022 to identify randomized controlled trials (RCTs) comparing immunostimulants administered by any method, with placebo to prevent ARTIs on children under 18 years of age without immunodeficiencies, anatomical, genetic, or allergic conditions. In order to analyze data from the studies, we used Review Manager 5.4 (The Cochrane Collaboration, 2020), assessed the certainty of the evidence with Grading of Recommendations, Assessment, Development and Evaluations (GRADE), and assessed the quality and risk of bias of the studies using the RoB tool 1.0. Further, outcomes were combined and analyzed using meta-analysis, subgroup analysis, and sensitivity analysis. Throughout the review, we included 72 placebo-controlled clinical trials involving 12,229 children. The meta-analyses, however, included only 38 studies (52.8%) with 4643 children (38% of the total) with data on mean number of ARTIs. These studies demonstrated a reduction in the ARTIs (MD –1.12 [95%CI –1.39 to −0.85]) and ratio of means of ARTIs (0.61 [95%CI 0.54–0.69]), corresponding to a percentage reduction of 39% (95%CI, 46%–31%) with moderate-quality data. Nevertheless, since there was considerable to substantial heterogeneity and bias was unclear in all domains in 32 out of 72 trials, the quality of the evidence for efficacy was deemed low. Only 14 trials reported adverse events.

The review indicates that immunostimulants reduce the incidence of ARTIs by 40% on average in susceptible children, despite low-quality evidence, heterogeneity, and the possibility of publication bias. However, further studies are needed to establish immunostimulants' safety and efficacy profiles.

This review was conducted without the support of any funding and has no registered number.

## Introduction

Most acute respiratory tract infections (ARTIs) are caused by viruses.^1^ Nevertheless, it is not possible to develop vaccines for each of the hundreds of possible pathogenic agents. As a result, specific immunization may not be the best method for preventing ARTIs. A good example is the introduction of the pneumococcal conjugate vaccine, which led to a decrease in carriage and invasive infections due to the vaccine serotypes. Still, some non-vaccine serotypes are becoming antibiotic-resistant.^2^, ^3^, ^4^

The Immunology Study Group of the Italian Paediatric Society defined recurrent respiratory infections based on local epidemiological studies. The following criteria required the absence of any underlying pathological condition (primary or secondary immunodeficiency, cystic fibrosis, malformations of the airways, immotile-cilia syndrome) explaining recurrent respiratory tract infections and the presence of 1 of the following 3 conditions: having more than 6 respiratory infections per year; having more than 1 respiratory infection during the autumn and winter seasons (from September to March in the northern hemisphere); and/or having more than 3 lower respiratory tract infections per year. Additionally, the study group considered the possibility that repeat infections are caused in part by social and environmental factors, such as daycare attendance, family size, air pollution, parental smoking, and dampness in the home.^5^

Thus, several clinical trials have studied non-specific measures for preventing ARTIs, including nutritional supplements such as vitamin A,^6^ vitamin C,^7^ vitamin D,^8^ and trace elements;^9^ preventive antibiotics;^10^ herbal extracts;^11^ xylitol;^12^ and the use of immunostimulants from synthetic sources^13^^,^^14^ or of bacterial origin.^13^^,^^15^^,^^16^ In addition, bacterial extracts and synthetic compo-unds are currently used in Europe and Latin America to prevent ARTIs.

Since there is information concerning the effects of immunostimulants, this review and meta-analysis aimed to evaluate and update (since 2006) the evidence regarding the efficacy and safety profile of immunostimulants as preventives for ARTIs in children based on scientific evidence by addressing the following PICOST: Population (children aged under 18 years of age susceptible to acute respiratory tract infections); Intervention (any immunostimulants); Comparison (placebo); Outcome (number of ARTIs per treatment group during the study period); Study (randomized controlled trials); and Time (Trials of 3–12 months duration published from January 1965 to January 10, 2022).

## Materials and methods

### Selecting criteria

#### Types of studies

We evaluated randomized controlled trials (RCTs) in which immunostimulants (administered by any method) were compared to a placebo to prevent ARTIs. The study excluded trials involving interferon inducers, vitamins, homeopathic and traditional remedies, and nutritional supplements.

#### Types of participants

Children under age of 18 were included. Children with immunodeficiencies, anatomical alterations, genetic conditions, asthma, allergies, atopy, or chronic respiratory diseases were excluded; asthma and allergic conditions were not included because their symptoms could be confounded with ARTIs.

#### Types of interventions

Any method of administering an immunostimulant to prevent ARTIs was investigated. It was considered that immunostimulants could be administered in the presence of active ARTI and concomitant therapies such as antipyretics and antibiotics.

#### Types of outcome measures

In a broader sense, ARTI was defined as the occurrence of several specific conditions, such as colds, influenza, tonsillitis, pharyngitis, bronchitis, and otitis media. We also considered physician diagnosis of ARTI and adverse events.

Since aetiological agents were not considered, there was no distinction between bacterial and viral ARTIs.

##### Primary and secondary outcomes

To assess efficacy, the primary outcome was the number of ARTIs per treatment group during the study period.

Secondary outcomes were the ratio of means of ARTIs by treatment group and the incidence of adverse events.

## Search methods

### Electronic searches

Our search was conducted on the Cochrane Central Register of Controlled Trials (CENTRAL) 2021, Issue 12, a part of the Cochrane Library, www.thecochranelibrary.com (accessed on 10 January 2022), which includes the ARI Group's Specialized Register, Pubmed (2011–10 January 2022), Embase (Elsevier) (2011–10 January 2022), Google Scholar (2011–10 January 2022), and Scopus (Elsevier) (2011–10 January 2022). A search for previous versions of this work covered a period from 1965 to 2006.^17^

### Searching other resources

Citation searches in Google Scholar and Scopus were conducted using identified articles as references. To identify additional studies, we searched the bibliographies of all included trials and those of relevant reviews. No language or publication restrictions were imposed. We also searched the WHO ICTRP website (http://www.who.int/ictrp/en/) and the National Institutes of Health's ClinicalTrials.gov site (http://www.ClinicalTrials.gov/.)

## Data collection and analysis

### Selection of studies

The review's authors (AB, BEDRN, JJLSM) independently searched for trials for inclusion and resolved differences through discussion. The screening process was duplicated without any pre-calibration. The data collected were extracted independently and duplicated by 2 review authors (BEDRN, JJLSM). Potential disagreements were resolved by reviewing the papers collectively. The review's authors were able to read Spanish and English papers, as well as retrieve data from German, French, and other Romance languages. Several studies reported the number of infections and the standard deviation (SD) or standard error (SE).

Review Manager 5.4 (Review Manager [RevMan] [Computer program] Version 5.4 of The Cochrane Collaboration, 2020) was used for data input and analysis.

### Assessment of risk of bias in included studies

We measured trial quality using seven domains:

1. Random sequence generation (selection bias).
2. Allocation concealment (selection bias).
3. Blinding (performance bias and detection bias).
4. Blinding of participants and personnel (performance bias).
5. Blinding of outcome assessment (detection bias).
6. Incomplete outcome data (attrition bias).
7. Selective reporting (reporting bias).

We assigned for each included trial a quality rating as high risk, low risk, or uncertain risk for the above domains, using the criteria outlined in the Cochrane Handbook for Systematic Reviews of Interventions.^18^

### Pre-specified harms outcomes

It was determined that the intervention had the potential to cause harm by increasing the incidence of adverse reactions and ARTIs.

### Data synthesis

Across the studies, outcomes were combined and analyzed using meta-analysis, subgroup analysis, and sensitivity analysis. Variables included in the subgroup analyses were bacterial immunostimulants and the trials with a sample size of more than 40. *A priori*, subgroup analyses were performed as they were relevant in previous versions of the present meta-analysis.^17^ Initially, the protocol was published in the Cochrane Database of Systematic Reviews 2004,^19^ which did not include a simple pooled analysis, allowing us to consider the characteristics of subgroups and individual studies and avoid the appearance of spurious or counterintuitive results. For the sensitivity analyses, the type of immunostimulants (D53, levamisole, OM-85, RU40171, and Thymomodulin), as well as the number of ARTIs in the control group as <2; 2 to <4; ≥4; ≥4 without the outlier were considered. Finally, homogeneity was assessed using the I^2^ test.

### GRADE and "summary of findings table"

In order to create a summary of the findings table, we used the following outcomes: number of ARTIs, the ratio of means of ARTIs, and adverse events experienced. We used the Grading of Recommendations, Assessment, Development and Evaluations (GRADE) criteria (study limitations, consistency of effect, imprecision, indirectness, and publication bias) to assess the certainty of the evidence related to the studies that contributed data to the meta-analyses, and assessed the quality and risk of bias of individual studies using the RoB tool 1.0.^20^ The method and recommendations described in Section 8.5 and Chapter 12 of the Cochrane Handbook for Systematic Reviews of Interventions^18^ were applied using GRADEpro GDT software (GRADEpro GDT [Computer program]. Version accessed on 13 July 2020. Hamilton, ON: McMaster University [developed by Evidence Prime], 2020. Available at gradepro.org.).

## Results

### Description of studies

#### Results of the search

**Fig. 1:**
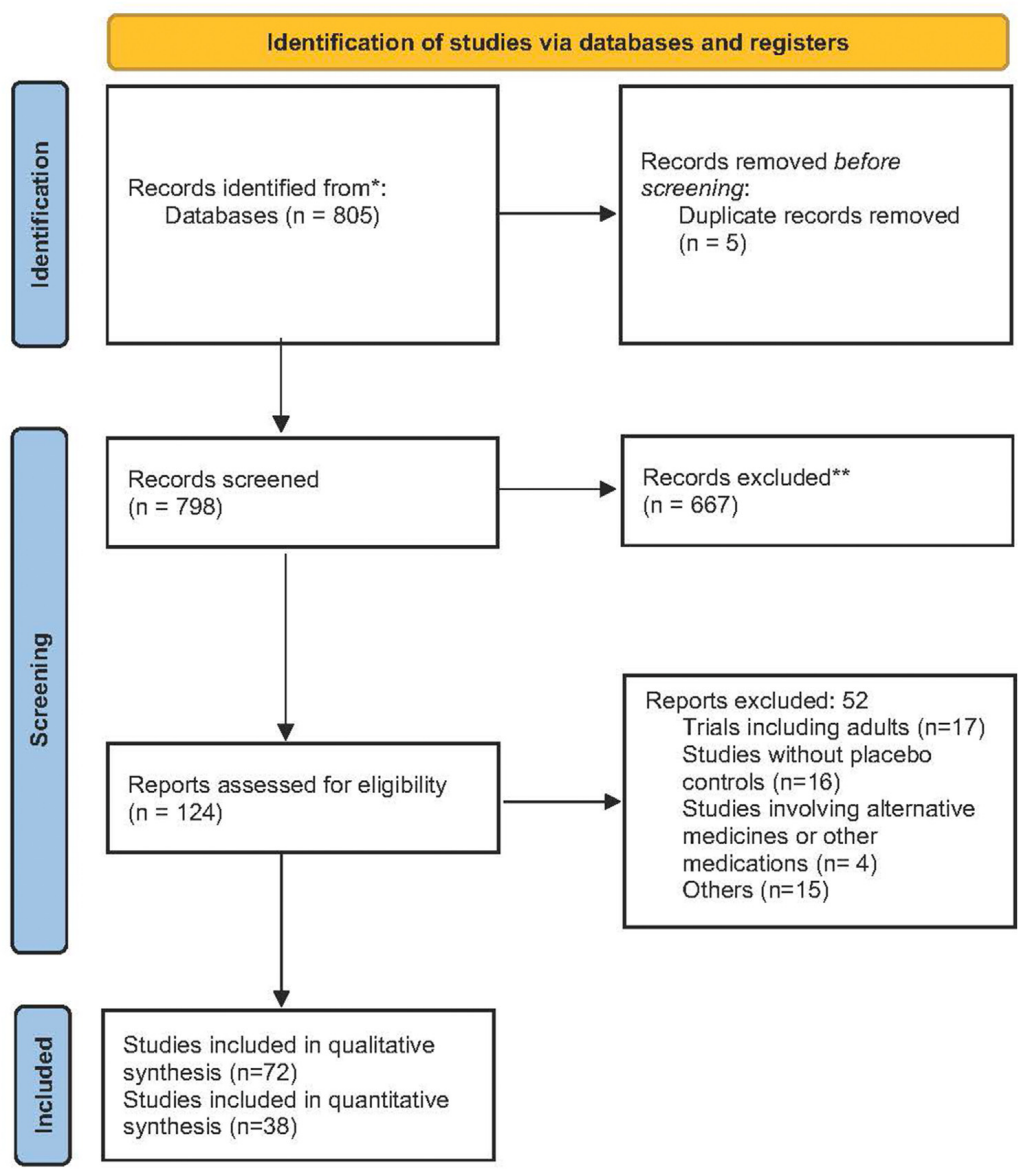
PRISMA flow diagram for search results and study selection

After searching electronic databases, we identified 798 references. However, only 124 studies were considered potentially relevant (see Fig. 1 “screening section” and supplementary material 1 and 2). No other potentially eligible studies were found through contact with trial authors or searches of trial registries.

#### Source of data

The data were obtained from the original research papers. Exceptions to this were data from OM-85 studies in Mexico;^21^, ^22^, ^23^, ^24^ data from Schaad et al,^25^ which were obtained from the review;^26^ and data from D53 studies.^27^, ^28^, ^29^, ^30^, ^31^

#### Included studies

A total of 72 placebo-controlled trials involving 12 229 children were included. There were diverse interventions, number of ARTIs in placebo groups, and outcomes reported in the included studies. We were only able to meta-analyze 38 studies (52.8% of the total) with 4643 children (38% of the total).

#### Population

In the included trials, participants ranged in age from 6 months to 18 years. The majority of the studies (n = 45) included children with recurrent ARTIs. Other trial participants (n = 4) had chronic or recurrent ARTIs. In some studies, participants were described as healthy or as having no significant health problems (n = 7). The rest of the studies included patients with acute and chronic infections or did not specify the patients' health status.

#### Settings

The most of the studies were conducted in paediatric practices, paediatric clinics, or subspecialty paediatric clinics. In 5 trials, schools or pre-schools were used as the setting.^32^, ^33^, ^34^, ^35^, ^36^ Other trials were conducted to some extent in nurseries or day-care centres (n = 3)^37^, ^38^, ^39^ In one study, participants lived in an orphanage (200 girls).^24^ The setting was not well defined or described in the remaining studies.

#### Interventions

Twenty-eight studies lasted less than 6 months, 37 studies lasted 6 months, and only 7 studies lasted more than 6 months. D53 trials lasted for less than 6 months in 5 cases, and 6 months in 15 cases. In the OM-85 trials, there was 1 study that lasted less than 6 months, 14 OM-85 trials lasted 6 months, and 2 trials lasted longer than 6 months. There was a lack of clarity regarding the methodology in all D53 trials, and they used different administration routes (orally or nasally).

### Outcomes

#### Multiple outcome data

Primary endpoints of the trials were diverse. The number of ARTIs, the percentage of children suffering more than one ARTI, the severity of the infection, and the number of days that the child had been ill were all included. Reports on these trials did not provide definitions of the end-points and the scales were not validated or consistent across authors. Consequently, we determined *a priori* that ARTIs, expressed as mean and standard deviation (SD), were the most appropriate choice for evaluating studies, as specified in the protocol and previous review.^19^ Studies reporting ARTIs as mean and SD also reported on other variables (eg, sick days, school absences, number of antibiotic treatments), which mirrored and were dependent upon the number of ARTIs.

The clinical scale results were not considered suitable for meta-analysis because the scales used were diverse, poorly described, and not validated.

Among the 72 included studies, only 38 reported the mean and SD of ARTIs or provided data to calculate these measures, allowing them to be included in the meta-analysis. These studies defined ARTIs based on respiratory symptoms and signs. The number of ARTIs during the longest observation period available was used. The remaining studies reported a variety of endpoints, including symptoms, clinical scale scores, or the presence and/or absence of respiratory infections (see Table 1).

**Table 1:** Description of studies not included in the meta-analysis according to the report of their results.

Characteristics of studies with median or mean number of ARTIs without SD or SE or a difference between them								
Author	Setting	Health status	Intervention	Outcomes	Treatment	Control	Reported P	Favored
Caramia 1994	Hospital-Clinic	Recurrent ARTIs	Pidotimod	Mean number of relapses	n = 60, 0.67	n = 60, 2.48	<0.001	Treatment
Carriere-Roussel 2017	Not specified	Recurrent ARTIs	D53	Median difference of ARTIs	n = 122, median difference −0.31 95% CI –0.18, −0.8	n = 132	<0.05	Treatment
Chen 2004	Paediatric Clinical Centre	Recurrent ARTIs	Lantigen B	Median of ARTIs	n = 37, 3	n = 37, 4	0.002	Treatment
Dils 1979	Not available	Chronic or recurrent ARTIs	Levamisole	Mean number of ARTIs	n = 45, 0.98	n = 41, 1.93	<0.001	Treatment
Fiocchi 1988	Paediatric Clinical Centre	Recurrent ARTIs	D53	Mean number of ARTIs	n = 30, 2.7	n = 30, 3.13	Not available	Not available
Longo 1988	Peadiatric Clinical Centre	Recurrent ARTIs	Thymomodulin	Mean number of ARTIs	n = 21, 1.24	n = 19, 3.79	<0.0002	Treatment
Passali 1994a	ENT centres	History tonsillitis or pharyngitis	Pidotimod	Mean number of ARTIs	n = 205, 1.54	n = 211, 2.63	<0.001	Treatment
Pozzi 2004	Not available	Recurrent ARTIs	Lantigen B	Mean number of ARTIs	n = 47, 1.211	n = 47, 1.643	Not available	Not available
Riedl-Seifert 1995	Paediatric Clinical Centre	Recurrent ARTIs	LW50020	Mean number of ARTIs	n = 99, 0.15	n = 108, 0.27	0.026	Treatment
Schaad 2010b	Not available	Recurrent ARTIs	OM-85	Mean number of ARTIs	n = 198, 1.97	n = 198, 2.42	0.0016	Treatment
Characteristics of the studies reporting clinical scores								
Fiocchi 1989	Paediatric clinical centre	Recurrent ARTIs	D53	Clinical score	n = 60, 4.2 ± 2.6	n = 58, 8.0 ± 4.3	<0.05	Treatment
Giovannini 2000	Paediatric clinical centre	Chronic or acute ARTIs	D53	Clinical score	n = 45, 0.46	n = 42, 0.76	<0.01	Treatment
Mora 2002	Not available	Recurrent ARTIs	D53	Clinical score	n = 41, not clear	n = 40, not clear	Not available	Not available
Mora 2007	ENT clinic	Recurrent ARTIs	D53	Clinical score	n = 80, 1.9	n = 80, 3.1	Not available	Not available
Renzo 2004	Not available	Chronic or recurrent ARTIs	D53	Clinical score	n = 36, 1.7	n = 36, 2.4	Not available	Not available
Characteristics of the studies reporting the presence or absence of ARTIs or Symptoms								
Burgio 1994	Not available	Recurrent ARTIs	Pidotimod	Presence respiratory symptoms	18%, 9/50	62.5%, 25/40	0.000	Treatment
Careddu 1994b	Not available	Recurrent ARTIs	Pidotimod	Presence of ARTIs	32%, 8/25	91.7%, 22/24	0.000	Treatment
Göhring 2017	Not available	Recurrent ARTIs	OM-85	Presence of ARTIs	84.6% 165/195	84.6% 170/201	0.889	No difference
Fukuda 1999	ENT clinic	Recurrent ARTIs	Thymomodulin	Presence of ARTIs	44.4%, 4/9	80%, 8/10	0.17	No difference
Mora 2010a	Not available	Recurrent ARTIs	D53	Presence of >1 acute adenoiditis	6.67%, 2/30	60%, 18/30	0.000	Treatment
Padayachee 2014	Pre-school children facilities	Healthy	Pelagonium	Presence of ARTIs	46.7%, 7/15	13.3%, 2/15	0.109	No difference
Paupe 1991	Clinics	Recurrent ARTIs	OM-85	Presence of ARTIs	60.7%, 37/61	83.7%, 46/55	0.011	Treatment
Rutishauser 1998	Not available	Recurrent ARTIs	LW50020	Presence of ARTIs	24.8%, 29/117	45.8%, 33/72	0.005	Treatment
Santamaria 2019	Paediatric pulmonology Clinics and paediatric office	Recurrent ARTIs	Pidotimod	Symptom days, % of total days	N = 13, 31%	N = 16, 56%	0.003	Treatment
Taylor 2003	Paediatric private practices	No significant health problems	Echinacea	Presence of >1 ARTIs	55.8%, 112/200	69.2%, 143/207	0.009	Treatment
Wahl 2008	Paediatric clinics and practices	Recurrent ARTIs	Echinacea	Presence of acute otitis	65%, 29/44	41%, 19/46	0.022	Control
Characteristics of the Studies Reporting Diverse Outcomes								
Andrianova 2003	Schools	Not defined	Allicor	ARTI morbidity	n = 42, reduced ARTI morbidity 1.7 fold compared to placebo	n = 41	<0.05	Treatment
Collet 1993	Day care centres	Healthy attending day care centre	OM-85	Presence of >4 upper ARTIs	26.7% 56/210 participants	33.8%, 72/213 with placebo	0.136	No difference
Espinosa Rosales 2009	Not available	Recurrent ARTIs	Pulmonarom	IL10 levels	n = 26, constant levels	n = 26, decreasing levels	0.034	Treatment
Fiocchi 2012	Day care centres/ENT clinic	Attending or to attend day-care-centre	D53	ARTI duration in days	n = 81, 3.6 ± 2.0	n = 77, 4.7 ± 2.5	0.04	Treatment; only a subgroup
Iuldashev 1988	Pre-school children institutions	Healthy children	Interferon	Infection rate of ARTIs	n = 1100, 1.3 fewer ARTIs than the placebo group.	n = 1078	0.05	Treatment subgroup
Mameli 2015	Family paediatricians	Healthy entering day-care, kinder	pidotimod	Infection rate of ARTIs	n = 29, 1.9 (95% CI 1.3 to 2.4)	n = 28, 2.4 (95% CI 1.8 to 3.0)	0.211	No difference
Martin du Pan 1982	Day nurseries, private practice	Day care attendance, susceptible to ARTTIs	OM-85	Days suffering purulent rhinorr-hoea	n = 36, 265/3660 days (7.24%)	n = 34, 569/3530 days (16.12%)	0.000	Treatment
Sramek 1986	Maternity School	Healthy and recurrent ARTIs	IRS19	ARTIs per 1000 persons days	n = 416, 7.79	n = 409, 7.43	>0.05	No difference

Twenty-two studies without sufficient data for meta-analysis supported immunostimulant treatment (including 2 studies that supported a subgroup treated with immunostimulant),^40^, ^41^, ^42^, ^43^, ^44^, ^45^, ^46^, ^47^, ^48^, ^49^, ^50^, ^51^, ^52^, ^53^, ^54^, ^55^, ^56^, ^57^, ^58^, ^59^, ^60^, ^61^ 6 studies showed no difference between immunostimulant and placebo groups,^62^, ^63^, ^64^, ^65^, ^66^, ^67^ and 5 studies did not explicitly report a statistical difference or superiority between groups.^34^^,^^68^, ^69^, ^70^, ^71^, ^72^ Only 1 study reported an increased incidence of ARTIs or related outcomes in immunostimulant-treated patients,^73^ as indicated in Table 1.

A total of 52 studies were excluded: 50 failed to meet the selection criteria, and 2 compared several immunostimulant treatments without a placebo group (see Table 2).

**Table 2:** Excluded studies the meta-analysis

Author, year	Reasons for their exclusion
Almeida, 1999	Participants with asthma were included
Banovcin, 1992	The trial was not double-blind or placebo-controlled
Barr, 1965	Trial with asthmatic children
Barrett, 2010	Children and adults were included
Braido 2014	Clinical trial with adults
Carlone, 2014	Clinical trial with adults
Colombo, 2014	Not a placebo-controlled trial
Das, 2000	Participants' ages were not specified
Doody-Oppikofer, 1998	The study examined only the acute phase of infection
Erman, 2009	A poorly defined homeopathic treatment
Fintelmann, 2012	Clinical trial with adults
Fontana, 1965	Clinical trial with children and adults
Graubaum, 2012	Clinical trial with adults
Grimfeld, 2004	An antihistamine was used in the trial
Grimm, 1999	Children and adults were not separated in the results
Heinz, 2010	Clinical trial with adults
Herrera-Basto, 1998	Researchers compared the effect of pidotimod only during the acute phase of ARTI
Jawad, 2012	Clinical trial with adults
Jesenak, 2013	Trials comparing vitamin C with placebo
Kondrat'eva, 2009	A poorly defined homeopathic treatment
Kozhukharova, 1987	The trial was not double-blind or placebo-controlled
Kudin, 2009	A poorly defined homeopathic treatment
Lauriello, 1990	Researchers compared the effect of the intervention only during the acute phase of ARTI
Lee, 2012	Clinical trial with adults
Licari, 2014	The trial was not double-blind or placebo-controlled
Luchikhin, 2000	The trial was not double-blind or placebo-controlled
Ma, 1994	The trial was not double-blind or placebo-controlled
Macchi, 2005	Clinical trial with adults
Makovetskaya, 2001	The trial was not double-blind or placebo-controlled
Mohammadi, 2014	Not a placebo-controlled trial
Mora, 2010b	A trial without the prevention approach for acute respiratory tract infections
Mueller, 1969	Participants with asthma were included
Namazova-Baranova, 2015	Not a placebo-controlled trial
Nespoli, 1992	Not a placebo-controlled trial
Obrosova-Serova, 1972	The trial was not double-blind or placebo-controlled
Oggiano, 1985	Open trial with children
Oldini, 1990	Children and adults were not separated in the results
Ortega del Alamo, 2005	Researchers compared the effect of the intervention only during the acute phase of ARTI
Predy, 2005	Clinical trial with adults
Prusek, 1987	Not a placebo-controlled trial
Razi, 2010	Participants with asthma were included
Rosaschino, 2004	Open trial
Rossi, 2004	Clinical trial with adults
Ruah, 2001	Not a placebo-controlled trial
Rytel, 1974	Clinical trial with adults
Scotti, 1987	Not a placebo-controlled trial
Sly PD, 2019	Only related to lower respiratory tract infections.
Steinsbekk, 2005	A poorly defined homeopathic treatment
Tiralongo, 2012	Clinical trial with adults
Vascotto, 1985	Not a placebo-controlled trial
Yale, 2004	Clinical trial with adults
Zagólski, 2015	Not a placebo-controlled trial

#### Risk of bias in included studies

**Fig. 2:**
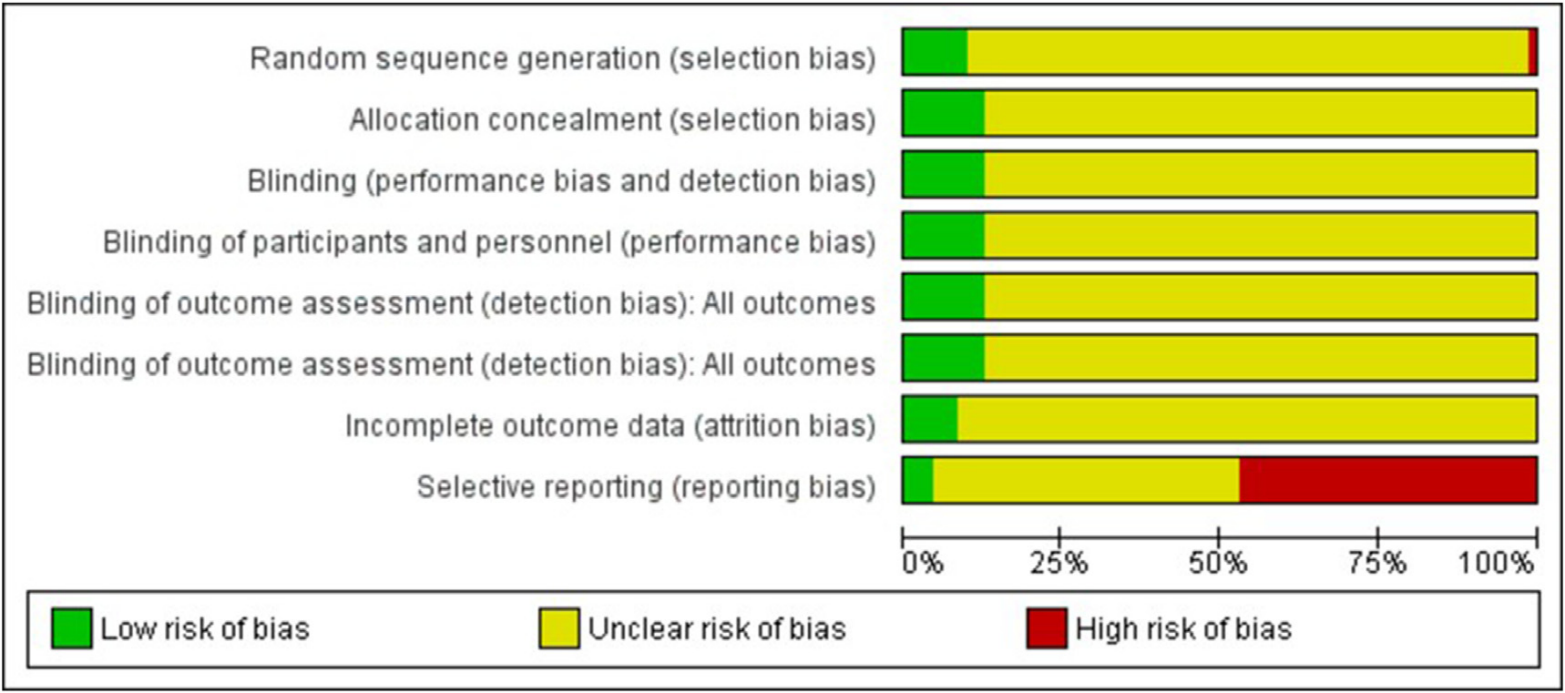
Summary of risk of bias in included studies according to GRADE

In 32 studies, bias risks were unclear in all domains. Allocation bias (selection bias) was low in 7 studies;^21^^,^^23^^,^^24^^,^^37^^,^^56^^,^^73^^,^^74^ blinding bias (performance bias and detection bias) was low in 8 studies;^21^, ^22^, ^23^, ^24^^,^^37^^,^^56^^,^^73^^,^^74^ incomplete outcome bias (attrition bias) was low in 5 studies;^21^^,^^23^^,^^24^^,^^73^^,^^56^ and selective reporting (reporting bias) was low in 3 studies.^21^^,^^23^^,^^73^ (See Fig. 2 and Supplementary material 3a,3b).

#### Primary outcome

**Fig. 3:**
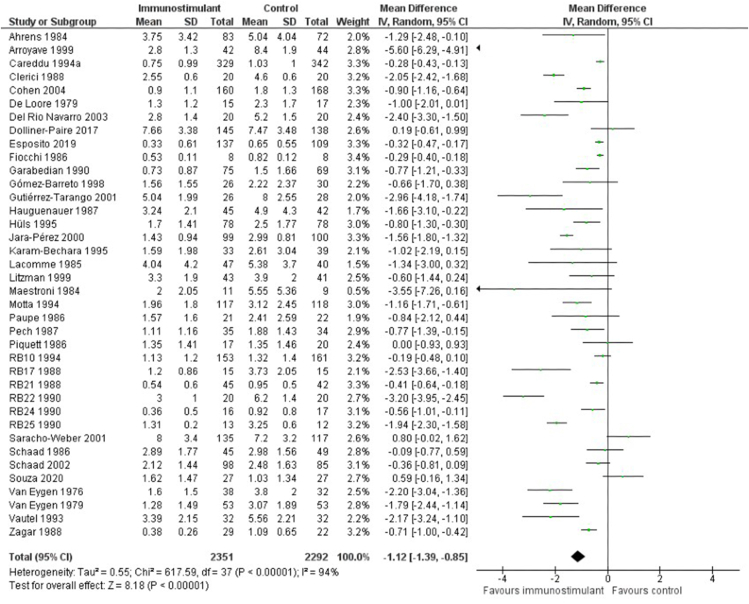
Mean difference of ARFs between the use of immunostimulants compared to placebo. Measures of heterogeneity (Tau^2^ and I^2^ statistics) and prediction intervals are also presented for the 38 studies analysis

As the primary outcome of the study was the number of ARTIs in children during the study period, comparing the use of immunostimulants with placebos showed to reduce the number of ARTIs with a mean difference (MD) of −1.12, 95% confidence interval (95%CI) −0.85, −1.39), see Fig. 3. The corresponding heterogeneity was I^2^ = 94%, Tau^2^ = 0.55; Chi^2^ = 617.59 and df = 37 (p < 0.00001). GRADE certainty of evidence (CoE) was moderate, but it was downgraded to low due to high bias and heterogeneity, so the quality of evidence was lower than expected. In addition, most studies failed to accurately report the incidence of adverse events. This led to an inadequate understanding of the safety profile of the intervention. See Table 3.

**Table 3:** Certainty of the evidence in the GRADE assessment of the effect of immunostimulant compared with placebo for preventing respiratory tract infection in children by the number of ARTIs, SD and incidence of adverse events.

Patient or population: children aged under 18 years of age susceptible to acute respiratory tract infections from clinics, private practices, hospital departments, schools, orphanages, etc. Intervention: Any immunostimulant with a trial period of 3–12 months. Comparison: Placebo O: Number of ARTIs per treatment group during the study period S: Randomized controlled trials T: Trials of 3–12 months duration published from January 1965 to January 10, 2022).					
Outcomes	Illustrative comparative risks' (95% CI)		Number of participants (studies)	Quality of the evidence (GRADE)	Comments
	Assumed risk	Corresponding risk			
	Placebo	Any immuno-stimulant			
Number of ARTIs	The range of ARTIs in the control group was 0.64–8.4	The mean number of ARTIs in the intervention groups was 1.12 lower (0.85–1.39 lower)	4643 (38 studies)	⊕⊕⊖⊖ low^a^	The heterogeneity depends on the number of ARTIs in the control group
Ratio of Means ARTIs		Ratio of means was 0.61 (95% CI 0.54, 0.69) corresponding to percentual reductions in ARTIS of 39% (31%–46%).	4643 (38 studies)	⊕⊕⊖⊖ low^a^	
Incidence of gastrointestinal adverse events	198/1276 (15.5%)	The odds ratio of adverse events regarding the intervention group was 0.93 (95% CI 0.65 to 1.33)	2565 (14 studies)	⊕⊖⊖⊖ very low^b^	Only 14 trials have a proper report of adverse events
Incidence of skin adverse events	28/1276 (2.2%)	The odds ratio of adverse events regarding the intervention group was 1.79 (95% CI 1.11 to 12.90)	2565 (14 studies)	⊕⊖⊖⊖ very low^b^	Only 14 trials have a proper report of adverse events

∗The basis for the assumed risk (e.g., the median control group risk across studies) is provided in footnotes. The corresponding risk (and its 95% CI) is based on the assumed risk in the comparison group and the relative effect of the intervention (and its 95% CI).

CI: confidence interval; RR: risk ratio; OR: odds ratio.

GRADE Working Group grades of evidence.

High quality: Further research is improbable to change our confidence in the estimate of effect.

Moderate quality: Further research is likely to have an important impact on our confidence in the estimate of effect and may change the estimate.

Low quality: Further research is very likely to have an important impact on our confidence in the estimate of effect and is likely to change the estimate.

Very low quality: We are very uncertain about the estimate.

a Heterogeneity was from considerable to substantial; the risk of bias was unclear for all the domains in 32 out of 72 trials. The quality of the evidence was downgraded from moderate to low.

b Adverse events were reported only in 14 trials implying selective outcome reporting. The quality of the evidence was downgraded from low to very low

#### Secondary outcomes

1. The ratio of means of ARTIs

**Fig. 4:**
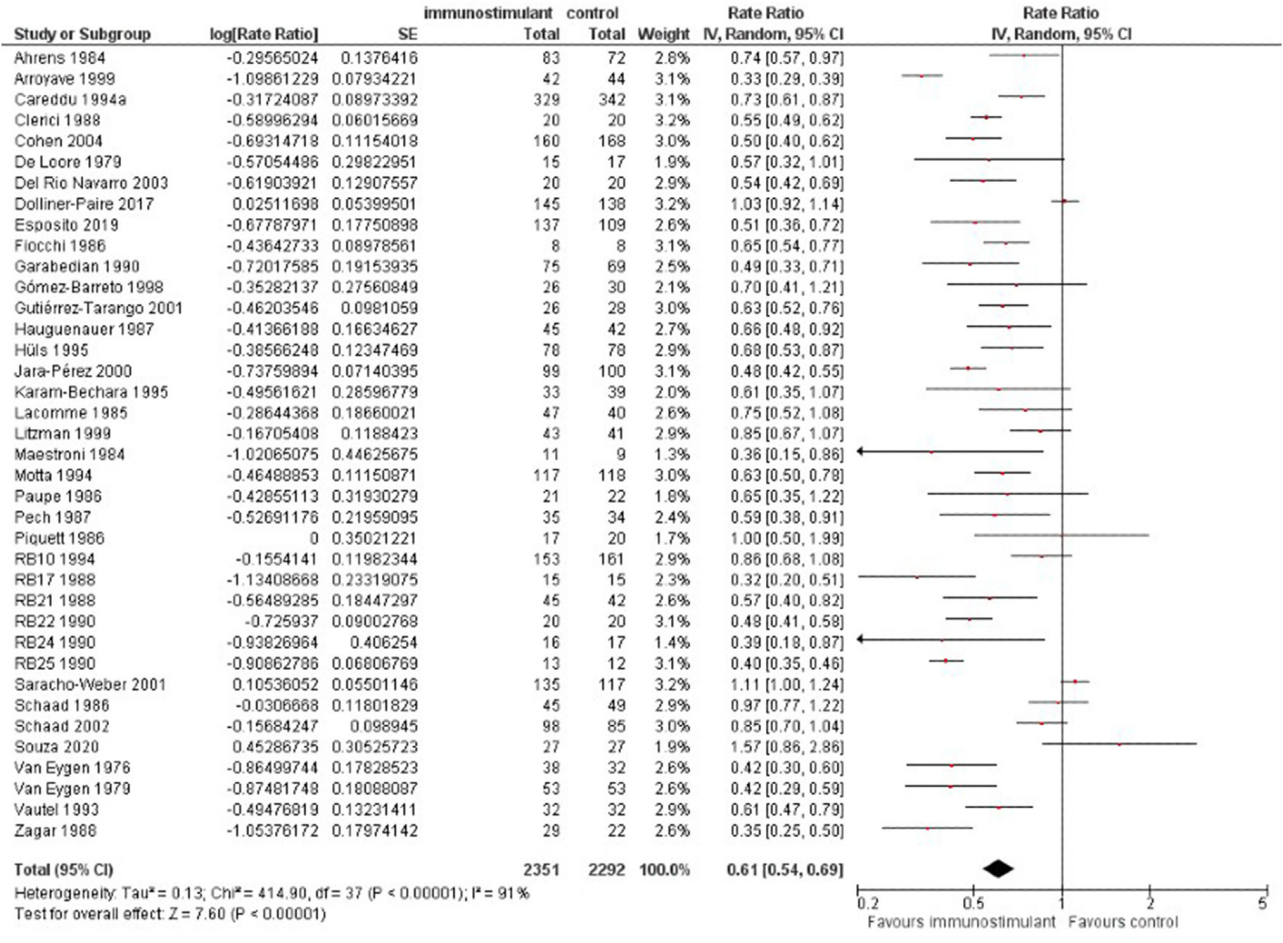
Rate radio of ARFs between the use of immunostimulants compared to placebo. Measures of heterogeneity (Tau^2^ and I^2^ statistics) and prediction intervals are also presented for the 38 studies analysis

ARTIs ratio mean was 0.61, (95% C,I 0.54–0.69), reflecting a percentual reduction of 39% (95%CI, 31–46) in the number of ARTIs. Heterogeneity: Tau^2^ = 0.13; Chi^2^ = 414.90, df = 37 (p = 0.00001) and I^2^ = 91%. GRADE CoE was moderate, but it was downgraded to low due to high bias and heterogeneity. See Fig. 4.

2. The incidence of adverse events

In total, 14 studies included in this meta-analysis reported adverse events, setting 2565 participants (1289 in the active treatment groups and 1276 in the placebo groups) for the gastrointestinal adverse events synthesis (nausea, vomiting, abdominal pain, and diarrhea) and 2565 participants (1289 in the active groups and 1276 in the placebo groups) for the skin adverse events synthesis. These were the most frequently reported adverse events (see supplemental material 2, “adverse events section”). The odds ratio for gastrointestinal adverse events was 0.93 (95% CI 0.65, 1.33). Heterogeneity: Tau^2^ = 0.07; Chi^2^ = 12.17, df = 9 (p = 0.20) and I^2^ = 26%. Test of overall effect: Z = 0.39 (p = 0.69) did not reveal a significant difference between groups. The odds ratio for adverse skin events was 1.79 (95% CI 1.11, 2.90). Heterogeneity: Tau^2^ = 0.00; Chi^2^ = 3.36, df = 6 (p = 0.76) and I^2^ = 0%. Test for overall effect: Z = 2.37 (p = 0.02) had a significant difference between groups, with more skin adverse events in the treatment group. GRADE CoE was low, but it was downgraded to very low as a result of inadequate reporting of adverse events in most of the trials.

### Other sub-group analyses

Several subgroup analyses were realized considering factors that could influence the results:

1. It included the data from bacterial immunostimulant studies (excluding the Saracho Weber trial,^75^ which was the only trial with more ARTIs in the immunostimulants group than the placebo group, possibly as a result of a clerical error inverting the ARTI incidences). In total, 27 trials were conducted with 2737 children, of whom 1400 received active treatment and 1337 received placebo treatment. ARTIs were reduced by MD -1.22 (95%CI -0.84,-1.60). Heterogeneity: Tau^2^ = 0.83; Chi^2^ = 448.97, df = 26 (p < 0.00001) and I^2^ = 94%. The ratio of means of ARTIs was 0.60 (95%CI 0.51, 0.71). Heterogeneity: Tau^2^ = 0.15; Chi^2^ = 280.62, df = 26 (p < 0.00001) and I^2^ = 91%.
2. Data from studies that involved at least 40 children and used bacterial immunostimulants (excluding the Saracho-Weber trial^75^). Twenty-two trials were conducted, involving 2592 children, with 1328 receiving immunostimulants and 1264 receiving placebo. The reduction in the total number of ARTIs was MD –1.19 (95% CI –0.77, −1.61). Heterogeneity: Tau^2^ = 0.83; Chi^2^ = 390.02, df = 21 (p < 0.00001); I^2^ = 95%. The ratio of means of ARTIs was 0.64 (95% CI 0.54, 0.75). Heterogeneity: Tau^2^ = 0.14; Chi^2^ = 225.36, df = 21 (p < 0.00001) and I^2^ = 91%.
3. Data from bacterial immunostimulants D53 and OM85 studies conducted with at least 40 children. There were 19 trials with 2394 participants, 1230 of whom received immunostimulants and 1164 took placebo. The reduction in the total number of ARTIs was MD –0.94 (95% CI –0.61, −1.28). Heterogeneity: Tau^2^ = 0.41; Chi^2^ = 190.38, df = 18 (p < 0.00001) and I^2^ = 91%. The ratio of means of ARTIs was 0.66 (95% CI 0.57, 0.77). Heterogeneity: Tau^2^ = 0.10; Chi^2^ = 146.91, df = 18 (p < 0.00001) and I^2^ = 88%.

## Sensitivity analyses

According to the Cochrane Manual:^76^ “A sensitivity analysis is a repeat of the primary analysis or meta-analysis, substituting alternative decisions or ranges of values for decisions that were arbitrary or unclear” and “some sensitivity analyses involve restricting the analysis to a subset of the totality of studies.” In addition to the subanalyses, the sensitivity analyses included the reduction in the total number of ARTIs considering the type of immunostimulants (D53, levamisole, OM-85, RU40171 and Thymomodulin), as well as the number of ARTIs in the control group (<2; 2 to <4; ≥4; ≥4 without the outliers) in all the subgroups (type of immunostimulant and the number of infections in the control group). The results for the difference in the mean number of ARTIs were similar, with the 95% CI overlapping (not statistically significant differences), except for the group with less than 2 ARTIs in the control group with lesser size of effect, indicating the robustness of the meta-analysis (see supplementary material 2).

## Discussion

Products with immunostimulant properties have been reported to activate immune cells with receptors that recognize common bacterial products or to provide additional stimulation to activate them.^77^ For instance, 2 bacterial lysates have been shown to activate TLR2,^78^^,^^79^ and levamisole may do the same.^80^ In another study, OM-85 induced interleukin-1beta, IL-6, and tumor necrosis factor alpha (TNF-α) in murine macrophages by activating TLR4 and TLR2 via the ERK1/2/NF kappa B pathway.^80^ Recent research suggests that OM-85 induces proIL-1 beta and proIL-1 alpha levels in bone marrow-derived dendritic cells without activating the inflammasome.^81^ On the other hand, the activation of the PI3K/Akt signaling pathway via the CXC Chemokine Receptor 3A (CXCR3A) isoform receptor is required for the adhesion and chemotaxis of monocytes induced by pidotimod, as well as the migration of activated T cells induced by IL-2.^82^

### Summary of main results

A relatively small number of papers met the standards for methodological quality and clinical trial reporting and the majority deviated significantly from these standards. Additionally, many of the trial publications lacked clarity, reducing the quality of the information.

Based on the current review, immunostimulants may be able to prevent ARTI. To establish the actual effects of immunostimulants and the effects of individual immunostimulant preparations, more extensive clinical trials should be conducted, with adequate power for important population groups and sponsored by health authorities.

### Overall completeness and applicability of evidence

It is possible that some studies with negative results have not been published due to the positive outcome results bias.^17^ In addition, the risk of bias is unclear for 32 studies in all domains, 34 studies had high risks for reporting bias, and 8 studies had low risks in some bias domains (see supplementary material 4).

### Quality of the evidence

In 32 out of 72 trials, the risk of bias was unclear for all domains. The quality of the evidence for the safety of the intervention has been downgraded from low to very low because adverse events were reported in only 14 trials, suggesting selective reporting. This is summarized in Table 3.

### Agreements and disagreements with other studies or reviews

This study supports a prior meta-analysis of the effects of immunostimulants, in which a percent decline in ARTIs was measured at 42.64% (95%IC, −40.08, −45.19).^83^

In a review of D53's effectiveness in reducing the incidence of ARTIs among children, it was also found to decrease ENT bronchopulmonary infections by 32%–61% in comparison to a placebo,^29^ which is consistent with the effect of D53 shown in this review.

Another meta-analysis of individual immunostimulants reports an ARTIs reduction of −31.86% (95%CI, −29.40, −34.32) for D53, and a corresponding reduction of −39.28% (95%CI, −25.98, −52.58) for OM-85.^84^ Both CIs are in agreement with those in this study. Based on one meta-analysis,^84^ 32% of the OM-85 treated patients experienced three or more ARTIs in 6 months, compared to 58.2% of the placebo-treated patients. With OM-85, the reduction was −1.21 (95%CI, −1.03, −1.39), similar to those found in this study.

This review's findings disagree with those published by Steurer-Stey,^26^ who pooled two OM-85 studies to calculate the risk of fewer than 3 infections over 6 months of follow-up in children not in daycare (risk ratio = 0.82 [95%CI, 0.65,1.02]).

In another meta-analysis, a single polyvalent mechanical bacterial lysate was examined. Multiple non-placebo studies in different age groups and indications were combined in this study. According to the results of a sub-analysis in three studies, which included 193 treated children and 153 untreated children, the ARTI rate was reduced by 2.2 (CI 95% 3.3 to 1.1).^85^ This is consistent with the findings of this study.

An extensive review of the efficacy and safety of OM-85 in children included both placebo-controlled studies published internationally, and uncontrolled studies conducted in China. The study found a reduction in ARTIs of −2.33 (95%CI –1.90, −2.75), P = 0.00001. Despite the fact that efficacy was greater in this study, adverse event rates were higher (RR 1.39 [95%CI 1.02, 1.88]; P = 0.04).^86^

In China, a systematic review of pidotimod in children, including placebo and non-placebo-controlled trials, was conducted. In the review, 24 studies were considered; 1912 patients were assigned to the pidotimod group, and 1848 patients were assigned to the conventional treatment group. The outcome was the proportion of children experiencing a relapse of ARTIs with a score of 0, 1, or 2. The proportion of participants who took pidotimod had fewer infections; the relative risk was 1.59, (95%CI, 1.45–1.74), I2 = 51%, p = 0.00001 compared to those who took conventional treatment. It is not possible to compare these efficacy findings with those of other meta-analyses. Pidotimod did not appear to increase the risk of adverse events statistically significantly.^87^

### Limitations

Using the most relevant databases, we identified and selected all potentially relevant references to other studies. We also examined articles citing all identified studies. Additionally, authors and manufacturers were contacted (see previous version of this review Del-Rio-Navarro 2012).^16^ However, this review has limitations because of the information quality, heterogeneity, and the possibility of publication bias.

We may have missed some studies because they were never published, published in obscure locations, rarely cited, or incorrectly indexed in databases. The publication bias of neutral, negative, uninteresting, or unwanted results in studies sponsored by pharmaceutical companies must be taken into account.

Although most of the studies (with ARTI as mean and dispersion) were integrated into the meta-analysis, other studies reporting different results were not included.

## Authors' conclusions

According to this review, immunostimulants reduce the incidence of ARTIs by 40% on average among susceptible children. Trial studies have shown the benefits of immunostimulants in toddlers (2–5 years of age), schoolchildren (6–12 years of age), and children with high incidences of ARTIs, such as those in daycare centers or orphanages. A further high-quality trial is required to confirm the true effect of immunostimulants and individual immunostimulant preparations on the prevention of ARTIs. We encourage national health authorities to conduct large, multicenter, double-blinded, placebo-controlled studies to establish the precise benefits and risks of using immunostimulants to prevent ARTIs.

## Abbreviations

ARTIs, acute respiratory tract infections; RCTs, randomized controlled trials; CI, confidence intervals; SD, standard deviation; MD, mean difference.

## Acknowledgments

We would like to acknowledge especially the outstanding editorial work of Liz Dooley and Vicki Flenady. Vicki Flenady was a co-author of earlier versions of this systematic review and San Francisco Edit (https://www.sfedit.net/) edited and proofread it. Finally, we wish to thank Axel Arturo Berber-Del-Rio for proofreading the last version of the manuscript.

## Funding

No financial support for this work that could have influenced its outcome.

## Availability of data and materials

The datasets used and/or analyzed during the current study are available from the corresponding author on reasonable request.

## Contributors’ statement page

Arturo Berber and Blanca Estela Del-Rio-Navarro: wrote the protocol, conducted the bibliographical search, extracted data for meta-analyses, realize the statistical analyses, and prepared the first draft, and final manuscript. She is the corresponding author.

Nayely Reyes-Noriega: drafted and revised the final version of the systematic review. She also contributed to the development of the graphics and supplementary information of this revision, as well as the interpretation of the results of the meta-analysis, subanalysis, and sensitivity analysis.

Juan José Luis Sienra-Monge: reviewed the protocol, conducted the bibliographical search, extracted data for meta-analyses and reviewed the final manuscript.

All authors approved the final manuscript as submitted and agree to be accountable for all aspects of the work.

## Ethics approval and consent to participate

The authors declare that all procedures were carried out in accordance with the ethical standards of the institutional committee on human investigation, the World Medical Association, and the Helsinki Declaration. Ethics committee review and patient consent were not required, as this was an investigation of the literature.

## Consent for publication

All authors consent this article for publication.

## Declaration of competing interest

The authors declare that they have no conflict of interest in relation to the methods or materials employed in this study.

## Cochrane registration

https://doi.org/10.1002/14651858.CD004974.pub2.

## Figures and Tables

**Multimedia component 3:**
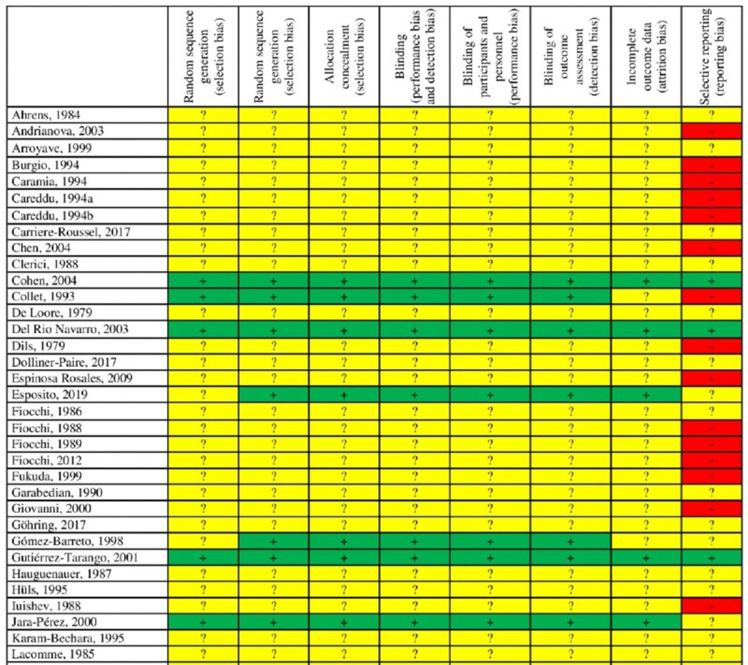


**Multimedia component 4:**
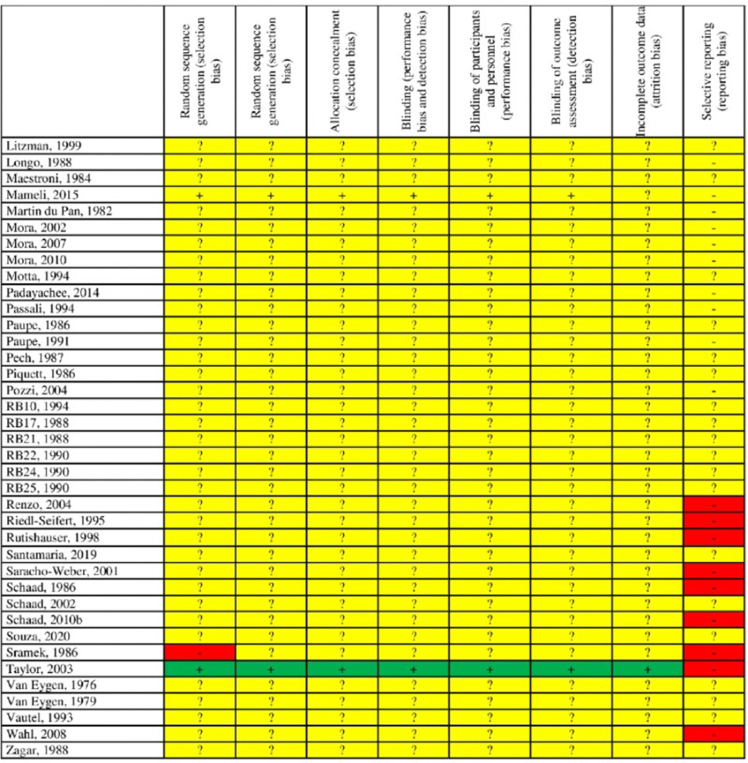


**Multimedia component 5:**