# Supplementary material for: Immunostimulants for preventing respiratory tract infection in children: A systematic review and meta-analysis
Source: World Allergy Organ J. 2022 Sep 14;15(9):100684. doi: 10.1016/j.waojou.2022.100684 (PMC9483654; doi:10.1016/j.waojou.2022.100684)
Supplement: Multimedia component 1 [file mmc1.docx]

## Supplementary material 1: List of study search strategies.

## 1.- Pubmed (Ovid) search strategy

1 exp Respiratory Tract Infections/
2 (respiratory adj5 infect*).tw.
3 (ari or arti or urti or lrti).tw.
4 Common Cold/
5 (common cold* or coryza).tw.
6 exp Otitis Media/
7 otitis media.tw.
8 (aom or ome).tw.
9 exp Pharyngitis/
10 (sore throat* or pharyngit* or rhinopharyngit* or nasopharyngit* or tonsillit* or pharyngotonsillit*).tw.
11 exp Sinusitis/
12 (sinusit* or rhinosinusit* or nasosinusit*).tw.
13 Influenza, Human/
14 (influenza* or flu).tw.
15 exp Bronchitis/
16 (bronchit* or bronchiolit* or tracheobronchit*).tw.
17 exp Laryngitis/
18 (laryngit* or croup or laryngotracheit* or laryngotracheobronchit*).tw.
19 exp Pneumonia/
20 (pneumon* or bronchopneumon* or pleuropneumon*).tw.
21 Sneezing/
22 Cough/
23 (sneez* or cough* or ((runny or running) adj1 nose*) or nasal congestion).tw.
24 or/1-23
25 exp Adjuvants, Immunologic/
26 immunostimulant*.tw,nm.
27 immunomodulat*.tw,nm.
28 immunoadjuvant*.tw,nm.
29 immunologic adjuvant*.tw,nm.
30 (immunobalt or lw50020 or luivac or paspat or munostin).tw,nm.
31 (om-85 bv or om85bv or om 85 bv).tw,nm.
32 (bronchovaxom or broncho-vaxom or broncho vaxom).tw,nm.
33 (pulmonar-om or pulmonar om).tw,nm.
34 d53.tw,nm.
35 (ribomunyl or ribovac or immucytal).tw,nm.
36 Lipopolysaccharides/
37 lipopolysaccharide*.tw,nm.
38 (ru41740 or ru-41740 or ru 41740 or biostim).tw,nm.
39 Thymus Extracts/
40 thymus extract*.tw,nm.
41 (thymic extract* or thymomodulin*).tw,nm.
42 Pelargonium/
43 (pelargonium* or umckaloabo).tw,nm.
44 (am3 or imunoferon or immunoferon or inmunoferon).tw,nm.
45 glycophosphopep*.tw,nm.
46 (pidotimod or adimod).tw,nm.
47 Levamisole/
48 levamisole.tw,nm.
49 or/25-48
50 24 and 49

## 2.- Embase (Elsevier) search strategy

#46 #44 AND #45
#45.8 #45.3 NOT #45.7
#45.7 #45.4 NOT #45.6
#45.6 #45.4 AND #45.5
#45.5 'human'/de
#45.4 'animal'/de OR 'nonhuman'/de OR 'animal experiment'/de
#45.3 #45.1 OR #45.2
#45.2 random*:ab,ti OR placebo*:ab,ti OR crossover*:ab,ti OR 'cross over':ab,ti OR allocat*:ab,ti OR trial:ti OR (doubl* NEXT/1 blind*):ab,ti
#45.1 'randomized controlled trial'/exp OR 'single blind procedure'/exp OR 'double blind procedure'/exp OR 'crossover procedure'/exp
#44 #23 AND #43
#43 #24 OR #25 OR #26 OR #27 OR #28 OR #29 OR #30 OR #31 OR #32 OR #33 OR #34 OR #35 OR #36 OR #37 OR #38 OR #39 OR #40 OR #41 OR #42
#42 levamisole:ab,ti
#41 pidotimod:ab,ti OR adimod:ab,ti
#40 glycophosphopep*:ab,ti
#39 am3:ab,ti OR imunoferon:ab,ti OR immunoferon:ab,ti OR inmunoferon:ab,ti
#38 pelargonium*:ab,ti OR umckaloabo:ab,ti
#37 'pelargonium sidoides extract'/de
#36 'thymus extract':ab,ti OR 'thymus extracts':ab,ti OR 'thymic extract':ab,ti OR 'thymic extracts':ab,ti OR thymomodulin*:ab,ti
#35 'thymus extract'/de AND [embase]/lim525
#34 ru41740:ab,ti OR 'ru-41740':ab,ti OR 'ru 41740':ab,ti OR biostim:ab,ti
#33 lipopolysaccharide*:ab,ti
#32 'lipopolysaccharide'/exp
#31 ribomunyl:ab,ti OR ribovac:ab,ti OR immucytal:ab,ti
#30 d53:ab,ti
#29 'pulmonar-om':ab,ti OR 'pulmonar om':ab,ti
#28 bronchovaxom:ab,ti OR 'broncho-vaxom':ab,ti OR 'broncho vaxom':ab,ti
#27 'om 85 bv':ab,ti OR om85bv:ab,ti OR 'om-85 bv':ab,ti
#26 immunobalt:ab,ti OR lw50020:ab,ti OR luivac:ab,ti OR paspat:ab,ti OR munostin:ab,ti
#25 immunostimulant*:ab,ti OR immunomodul*:ab,ti OR immunoadjuvant*:ab,ti OR (immuno* NEAR/1 adjuvant*):ab,ti
#24 'immunological adjuvant'/exp OR 'immunostimulating agent'/exp
#23 #1 OR #2 OR #3 OR #4 OR #5 OR #6 OR #7 OR #8 OR #9 OR #10 OR #11 OR #12 OR #13 OR #14 OR #15 OR #16 OR #17 OR #18 OR #19 OR #20 OR #21 OR #22
#22 sneez*:ab,ti OR cough*:ab,ti OR (runny OR running) NEAR/1 nose* OR 'nasal congestion':ab,ti
#21 'sneezing'/de OR 'coughing'/de
#20 pneumon*:ab,ti OR bronchopneumon*:ab,ti OR pleuropneumon*:ab,ti
#19 'pneumonia'/de OR 'bronchopneumonia'/de OR 'infectious pneumonia'/exp
#18 laryngit*:ab,ti OR croup:ab,ti OR laryngotracheit*:ab,ti
#17 'laryngitis'/de OR 'croup'/de
#16 bronchit*:ab,ti OR bronchiolit*:ab,ti OR tracheobronchit*:ab,ti
#15 'bronchitis'/de OR 'bronchiolitis'/de OR 'laryngotracheobronchitis'/de OR 'tracheobronchitis'/de
#14 influenza*:ab,ti OR flu:ab,ti
#13 'influenza'/exp
#12 sinusit*:ab,ti OR rhinosinusit*:ab,ti OR nasosinusit*:ab,ti
#11 'sinusitis'/de OR 'acute sinusitis'/de OR 'bacterial sinusitis'/de OR 'viral sinusitis'/de
#10 'sore throat':ab,ti OR 'sore throats':ab,ti OR pharyngit*:ab,ti OR nasopharyngit*:ab,ti OR rhinopharyngit*:ab,ti OR pharyngotonsillit*:ab,ti OR tonsillit*:ab,ti
#9 'sore throat'/de
#8 'pharyngitis'/de OR 'rhinopharyngitis'/de OR 'viral pharyngitis'/de
#7 'otitis media':ab,ti OR aom:ab,ti OR ome:ab,ti
#6 'otitis media'/de OR 'acute otitis media'/exp
#5 'common cold':ab,ti OR 'common colds':ab,ti OR coryza:ab,ti
#4 'common cold'/de OR 'common cold symptom'/de
#3 ari:ab,ti OR arti:ab,ti AND urti:ab,ti OR lrti:ab,ti
#2 (respiratory NEAR/5 infect*):ab,ti
#1 'respiratory tract infection'/de OR 'upper respiratory tract infection'/de OR 'viral upper respiratory tract infection'/de OR 'lower respiratory tract infection'/de

## 3.- Google Scholar search strategy

((immunostimulant OR immunomodulator OR immunoadjuvant OR immunologic adjuvant OR immunoblat OR lw50020 OR luivac OR paspat OR munostin OR om-85 bv OR om85bv OR om 85 bv OR broncho vaxom OR broncho-vaxom OR broncho vaxom)) AND placebo AND respiratory

((pulmonar-om OR pulmonarcom OR d53 OR ribomunyl OR ribovac OR immucytal OR ru41740 OR ru-41740 OR ru 41740 OR biostim OR thymus extract OR thymic extract OR thymomodulin)) AND placebo AND respiratory

((pelargonium OR umckaloabo OR am3 OR immunoferon OR immunoferon OR inmunoferon OR glycol phosphopep OR pidotimod OR adimond OR levamisole)) AND placebo AND respiratory

## 4.- Scopus (Elsevier) search strategy

Search ((immunostimulant OR immunomodulator OR immunoadjuvant OR immunologic adjuvant OR immunoblat OR lw50020 OR luivac OR paspat OR munostin OR om-85 bv OR om85bv OR om 85 bv OR broncho vaxom OR broncho-vaxom OR broncho vaxom OR pulmonar-om OR pulmonarcom OR d53 OR ribomunyl OR ribovac OR immucytal OR ru41740 OR ru-41740 OR ru 41740 OR biostim OR thymus extract OR thymic extract OR thymomodulin OR pelargonium OR umckaloabo OR am3 OR immunoferon OR immunoferon OR inmunoferon OR glycolphosphopep OR pidotimod OR adimond OR levamisole)) AND respiratory Filters: Clinical Trial; published in the last 5 years

## 5.- Previous searches

For the 2011 version of the review we searched the Cochrane Central Register of Controlled Trials (CENTRAL) 2011, issue 1, which contains the Acute Respiratory Infections Group's Specialised Register, Pubmed (1966 to February week 4, 2011), EMBASE (1990 to February 2011), Google Scholar (2009 to February 2011), Scopus (2009 to February 2011), PASCAL (1990 to February 2010), SciSearch (1990 to February 2010) and IPA (1990 to February 2010). The search strategies used are available in the 2011 version of the review.^53^

For the 2006 version of the review we searched the Cochrane Central Register of Controlled Trials (CENTRAL) (The Cochrane Library Issue 4, 2005); Pubmed (January 1966 to January 2006); EMBASE (January 1990 to January 2006); PASCAL (up to January 2006); SciSearch (up to January 2006); and IPA (up to January 2006) for reports of trials. The search strategies used are available in the 2006 version of the review.^52^
