# Supplementary material for: Immunostimulants for preventing respiratory tract infection in children: A systematic review and meta-analysis
Source: World Allergy Organ J. 2022 Sep 14;15(9):100684. doi: 10.1016/j.waojou.2022.100684 (PMC9483654; doi:10.1016/j.waojou.2022.100684)
Supplement: Multimedia component 2 [file mmc2.docx]

# Supplementary material 2: Meta-analysis complete results

## 1.- Any immunostimulant compared with placebo.

| Outcome or Subgroup | Studies | Participants | Statistical Method | Effect Estimate |
| --- | --- | --- | --- | --- |

| 1.1 Mean number of ARTIs | 38 | 4643 | Mean Difference (IV, Random, 95% CI) | -1.12 [-1.39, -0.85] |
| --- | --- | --- | --- | --- |
| 1.2 Mean number of ARTIs in D53 group | 11 | 1067 | Mean Difference (IV, Random, 95% CI) | -1.32 [-1.86, -0.79] |

| 1.3 Mean number of ARTIs in levamisole group | 3 | 208 | Mean Difference (IV, Random, 95% CI) | -1.72 [-2.32, -1.12] |
| --- | --- | --- | --- | --- |
| 1.4 Mean number of ARTIs in OM-85 group | 12 | 1435 | Mean Difference (IV, Random, 95% CI) | -0.83 [-1.29, -0.36] |

| 1.5 Mean number of ARTIs in RU40171 group | 5 | 487 | Mean Difference (IV, Random, 95% CI) | -1.29 [-3.75, 1.17] |
| --- | --- | --- | --- | --- |
| 1.6 Mean number of ARTIs in Thymomodulin group | 3 | 128 | Mean Difference (IV, Random, 95% CI) | -1.12 [-2.51, 0.27] |

| 1.7 Mean number of ARTIs with less than 2 infections in the control group | 12 | 2050 | Mean Difference (IV, Random, 95% CI) | -0.43 [-0.58, -0.28] |
| --- | --- | --- | --- | --- |
| 1.8 Mean number of ARTIS with 2 to less than 4 infections in the control group | 14 | 1385 | Mean Difference (IV, Random, 95% CI) | -1.18 [-1.55, -0.81] |

| 1.9 Mean number of ARTIS with 4 or more infections in the control group | 12 | 1208 | Mean Difference (IV, Random, 95% CI) | -2.05 [-3.13, -0.98] |
| --- | --- | --- | --- | --- |
| 1.10 Mean number of ARTIs with 4 or more infections in th control group without Saracho-Weber 2001.^50^ | 11 | 956 | Mean Difference (IV, Random, 95% CI) | -2.33 [-3.35, -1.32] |

| 1.11 Ratio of Means of ARTIs | 38 | 4643 | Rate Ratio (IV, Random, 95% CI) | 0.61 [0.54, 0.69] |
| --- | --- | --- | --- | --- |
| 1.12 Ratio of Means of ARTIs with less than 2 infections in the control group | 12 | 2050 | Rate Ratio (IV, Random, 95% CI) | 0.61 [0.51, 0.73] |

| 1.13 Ratio of Means of ARTIs with 2 to less than 4 infections in the control group | 14 | 1385 | Rate Ratio (IV, Random, 95% CI) | 0.59 [0.48, 0.71] |
| --- | --- | --- | --- | --- |
| 1.14 Ratio of Means of ARTIs with 4 or more infections in the control group | 12 | 1208 | Rate Ratio (IV, Random, 95% CI) | 0.63 [0.49, 0.81] |

| 1.15 Ratio of Means of ARTIs with 4 or more infections in the control group without Saracho-Weber 2001.^50^ | 11 | 956 | Rate Ratio (IV, Random, 95% CI) | 0.59 [0.46, 0.76] |
| --- | --- | --- | --- | --- |

## 2.- Adverse events.

| Outcome or Subgroup | Studies | Participants | Statistical Method | Effect Estimate |
| --- | --- | --- | --- | --- |
| 2.1 Gastrointestinal adverse events | 14 | 2565 | Odds Ratio (M-H, Random, 95% CI) | 0.93 [0.65, 1.33] |
| 2.2 Skin adverse events | 14 | 2565 | Odds Ratio (M-H, Random, 95% CI) | 1.79 [1.11, 2.90] |

## 3.- Bacterial immunostimulants compared with placebo.

| Outcome or Subgroup | Studies | Participants | Statistical Method | Effect Estimate |
| --- | --- | --- | --- | --- |
| 3.1 Mean number of ARTIs | 27 | 2737 | Mean Difference (IV, Random, 95% CI) | -1.22 [-1.60, -0.84] |
| 3.2 Ratio of Means of ARTIs | 27 | 2737 | Rate Ratio (IV, Random, 95% CI) | 0.60 [0.51, 0.71] |

## 4.- Bacterial immunostimulant trials with N => 40 compared with placebo.

| Outcome or Subgroup | Studies | Participants | Statistical Method | Effect Estimate |
| --- | --- | --- | --- | --- |
| 4.1 Mean number of ARTIs | 22 | 2592 | Mean Difference (IV, Random, 95% CI) | -1.19 [-1.61, -0.77] |
| 4.2 Ratio of Means of ARTIs | 22 | 2592 | Rate Ratio (IV, Random, 95% CI) | 0.64 [0.54, 0.75] |

## 5.- Bacterial immunostimulant trials with N => 40 only OM-85 and D53 compared with placebo.

| Outcome or Subgroup | Studies | Participants | Statistical Method | Effect Estimate |
| --- | --- | --- | --- | --- |
| 5.1 Mean number of ARTIs | 19 | 2394 | Mean Difference (IV, Random, 95% CI) | -0.94 [-1.28, -0.61] |
| 5.2 Ratio of Means of ARTIs | 19 | 2394 | Rate Ratio (IV, Random, 95% CI) | 0.66 [0.57, 0.77] |
